# Review of the Role of the Brain in Chemotherapy-Induced Peripheral Neuropathy

**DOI:** 10.3389/fmolb.2021.693133

**Published:** 2021-06-11

**Authors:** Maryam Omran, Elizabeth K. Belcher, Nimish A. Mohile, Shelli R. Kesler, Michelle C. Janelsins, Andrea G. Hohmann, Ian R. Kleckner

**Affiliations:** ^1^ University of Rochester Medical Center, Rochester, NY, United States; ^2^ The University of Texas at Austin, Austin, TX, United States; ^3^ Psychological and Brain Sciences, Program in Neuroscience and Gill Center for Biomolecular Science, Indiana University Bloomington, Bloomington, IN, United States

**Keywords:** chemotherapy, neuropathy, brain, clinical, translational

## Abstract

Chemotherapy-induced peripheral neuropathy (CIPN) is a common, debilitating, and dose-limiting side effect of many chemotherapy regimens yet has limited treatments due to incomplete knowledge of its pathophysiology. Research on the pathophysiology of CIPN has focused on peripheral nerves because CIPN symptoms are felt in the hands and feet. However, better understanding the role of the brain in CIPN may accelerate understanding, diagnosing, and treating CIPN. The goals of this review are to (1) investigate the role of the brain in CIPN, and (2) use this knowledge to inform future research and treatment of CIPN. We identified 16 papers using brain interventions in animal models of CIPN and five papers using brain imaging in humans or monkeys with CIPN. These studies suggest that CIPN is partly caused by (1) brain hyperactivity, (2) reduced GABAergic inhibition, (3) neuroinflammation, and (4) overactivation of GPCR/MAPK pathways. These four features were observed in several brain regions including the thalamus, periaqueductal gray, anterior cingulate cortex, somatosensory cortex, and insula. We discuss how to leverage this knowledge for future preclinical research, clinical research, and brain-based treatments for CIPN.

## Introduction

Chemotherapy-induced peripheral neuropathy (CIPN) is a highly prevalent and severe toxicity of many widely used chemotherapy drugs including platinum-based agents (oxaliplatin, cisplatin, carboplatin), taxanes (paclitaxel, docetaxel), vinca alkaloids, proteasome inhibitors, and thalidomide analogues (Staff et al., 2017; Chan et al., 2019). These neurotoxic anti-cancer agents are used to treat breast, lung, cervical, prostate, ovarian, testicular, gastrointestinal, and blood or bone marrow cancers. CIPN is a dose-limiting toxicity, meaning that it can result in dose interruptions, subtherapeutic dosing, or discontinued therapy, in turn negatively impacting cancer progression (Lyman, 2009). Acute symptoms of CIPN appear in the hours and days after an infusion (Reeves et al., 2012; Argyriou et al., 2013; Pachman et al., 2015) whereas persistent symptoms occur in approximately 68% of patients one month following completion of chemotherapy and 30% of patients five months later (Seretny et al., 2014). On average, patients with CIPN require 12 more outpatient visits, three more hospital days, and $17,000 USD more in medical expenses than matched patients without CIPN (Pike et al., 2012). Thus, CIPN can severely impair physical, social, emotional, functional, financial, and occupational aspects of life.

We use the term CIPN to encompass patient-reported symptoms, clinical signs, and mechanistic features (e.g., neurobiological factors that cause or exacerbate CIPN), as opposed to a narrower definition referring only to the damage, dysfunction, and death of peripheral neurons. The symptoms of CIPN are primarily felt in the hands and feet with some combination of numbness, tingling, shooting or stabbing pain, burning pain, cramping, and hypersensitivity to cold temperatures (e.g., cold weather, touching something cold) (Staff et al., 2017; Chan et al., 2018). The clinical signs and symptoms include loss of tactile or vibration sensitivity, cold-induced pain in the hands, feet, mouth, and throat (cold allodynia), changes in walking gait, weakness, loss of balance, orthostatic hypotension, and sometimes changes in peripheral sensory nerve conduction (e.g., reduced sensory nerve action potential amplitudes) (Staff et al., 2017). The mechanistic features putatively include loss of intraepidermal nerve fibers, mitochondrial dysfunction, neuroinflammation, oxidative stress, and other features mentioned below (Flatters et al., 2017; Chan et al., 2019; Zajaczkowska et al., 2019). There is no gold standard assessment for identifying CIPN, but its diagnosis depends on patient history, symptoms, neurologic examination and type and dose of chemotherapy (Loprinzi et al., 2020; Wasilewski and Mohile, 2020).

There are only minimally effective methods to treat or prevent CIPN despite over 20 years of research and nearly 100 clinical trials in humans (Hershman et al., 2014; Loprinzi et al., 2020). In fact, the only recommended treatment is the drug duloxetine (Loprinzi et al., 2020), which only mildly improves CIPN pain (Smith et al., 2013). There are also several promising yet unproven interventions to treat or prevent CIPN, such as exercise (Kleckner et al., 2021a; Kleckner et al., 2021b), acupuncture, scrambler therapy (peripheral nerve stimulation), cryotherapy, cannabinoids, and tricyclic antidepressants (Loprinzi et al., 2020). A recent report from the 2017 National Cancer Institute Clinical Trials Planning Meeting on CIPN concluded that the lack of effective CIPN treatments is partly due to an incomplete understanding of the pathophysiological mechanisms of CIPN (Dorsey et al., 2019). Therefore, herein we investigate a novel perspective on the pathophysiology of CIPN by focusing on the role of the brain in CIPN, as opposed to the peripheral nervous system.

The majority of research on CIPN mechanisms has focused on primary afferents of the peripheral nervous system. This rapidly growing body of research is rigorous and utilizes a variety of preclinical non-human animal models of CIPN. Typically, this involves rats or mice without cancer who repeatedly receive chemotherapy (usually oxaliplatin or paclitaxel) across several days or weeks to mimic how chemotherapy is delivered to human patients with cancer. This is combined with assessments of clinical signs of CIPN in the paws such as cold allodynia, mechanical allodynia, and mechanical hyperalgesia (Bonhof et al., 2019). Collectively, this research implicates multiple mechanisms documenting how chemotherapy causes peripheral nerve damage, dysfunction, and death (Flatters et al., 2017) including: (1) altered expression of ion channels and receptors that cause neuronal hyperactivity, (2) the innate immune response and inflammation, (3) mitochondrial dysfunction, and (4) changes in cell-signaling pathways such as G-coupled protein receptors (GPCRs) and mitogen-activated protein kinases (MAPK; see Table 1 for more details and citations). These are just some of the known mechanisms studied at the peripheral and spinal nerve levels in relation to CIPN, and other mechanisms likely contribute as well. Some of these same mechanisms extend to the brain (e.g., hyperactivity, inflammation, GPCR) with some important differences (e.g., the role of large-scale brain networks). Moreover, peripheral pathology seen in CIPN can lead to maladaptive responses in the brain that contribute to CIPN even if chemotherapy drugs do not enter the brain, as we discuss below. We postulate that knowledge of both peripheral and brain-based mechanisms can more holistically advance the study of CIPN.

**TABLE 1:** Overview of key pathways in the peripheral nerves implicated in CIPN.

Pathway	Details
Ion channels and receptors	CIPN appears to be caused by altered expression of ion channels and receptors, which lead to changes in neural activity (e.g., hyperactivity). For example, oxaliplatin causes prolonged opening of sodium channels (Grolleau et al., 2001; Webster et al., 2005); potassium channels are down-regulated in peripheral and dorsal root ganglia (DRG) nerves in CIPN (Descoeur et al., 2011; Thibault et al., 2012; Zhang and Dougherty, 2014); calcium channel expression is increased in the DRG after paclitaxel, and calcium channel antagonists (e.g., gabapentin) reduce CIPN symptoms in rodents (but not humans) (Flatters and Bennett, 2004; Xiao et al., 2007); CIPN has been associated with increases in expression of TRPV1 (heat-activated) in the DRG (Ta et al., 2010; Hara et al., 2013; Quartu et al., 2014), TRPA1 (cold-activated) expression (Nassini et al., 2011; Zhao et al., 2012), and TRPM8 (mild cold-activated)
Innate immune system and inflammation	The innate immune response and inflammation play a role in CIPN. For instance, the toll-like receptor-4 (TLR4), which is activated by bacterial pathogens, is also activated in the spinal cord in response to chemotherapy (Byrd-Leifer et al., 2001). CIPN symptoms can be reduced or prevented by blocking the TLR4 pathway during chemotherapy by way of an antagonist (Li et al., 2014; Li et al., 2015) or a genetic knockout (Park et al., 2014). Macrophages and inflammatory mediators such as CCL2, IL-1β, and TNF-α are all increased in the DRG during the development of CIPN (Woolf et al., 1997; Binshtok et al., 2008; Zhang et al., 2013; Zhang et al., 2016). These pro-inflammatory mediators cause neuronal hyperexcitability (Sorkin et al., 1997; Onda et al., 2002; Özaktay et al., 2002) by suppressing GABA production and glutamate clearance by spinal astrocytes
Mitochondrial dysfunction	Multiple studies have shown that paclitaxel, docetaxel, and oxaliplatin cause swollen and vacuolated mitochondria (Flatters and Bennett, 2006; Zhao et al., 2012; Zheng et al., 2012) with reduced respiration and ATP production (Zheng et al., 2011; Zheng et al., 2012) in peripheral sensory nerves and the DRG of the spinal cord. Second, oxidative stress is another hypothesis for CIPN development, as mitochondria and other cellular components are major sources of reactive oxygen species (ROS) and reactive nitrogen species (RNS) (Waseem et al., 2018). Both ROS and RNS affect neuronal excitability (Gamper and Ooi, 2015), and multiple studies have shown that various ROS scavengers reduce CIPN symptoms from paclitaxel (Kim et al., 2010; Fidanboylu et al., 2011, Janes et al., 2013)
Cell signaling pathways including GPCRs and MAPK	Changes in cell structural integrity (e.g., paclitaxel disrupting microtubules) and cell signaling pathways (e.g., G-coupled protein receptors [GPCRs], protein kinase C [PKC] (Chen et al., 2011), mitogen-associated protein kinase [MAPK] (Scuteri Galimberti et al., 2010) can lead to changes in neuronal growth including apoptosis. Some of these are linked to other above-mentioned pathways such as MAPK signaling as resulting from inflammation contributing to paclitaxel induced CIPN (Li et al., 2015)

Unfortunately, mechanism-based treatments for CIPN have not yet translated into many effective treatments in humans (Hu et al., 2019). For example, acetyl-l carnitine was a promising agent that reduced CIPN and improved peripheral nerve function in rodents via known effects on mitochondria (De Grandis, 2007), but a phase III randomized controlled trials (RCTs) in 409 patients found acetyl-l-carnitine worsened CIPN in humans (De Grandis, 2007; Hershman, Unger et al., 2013). A similar pattern was observed with the drug pregabalin, which successfully reduced CIPN in rodents by binding to voltage-gated calcium ion channels (Peng et al., 2012; Aoki et al., 2014), which are over-expressed in dorsal root ganglia (DRG) in CIPN (Gauchan et al., 2009). However, pregabalin failed to significantly reduce CIPN in a 199-patient randomized controlled trial in humans (De Andrade et al., 2017). Given the current lack of effective human treatments for CIPN derived from knowledge of peripheral pathways, research on CIPN needs a paradigm shift to focus on novel mechanisms.

**FIGURE 1:**
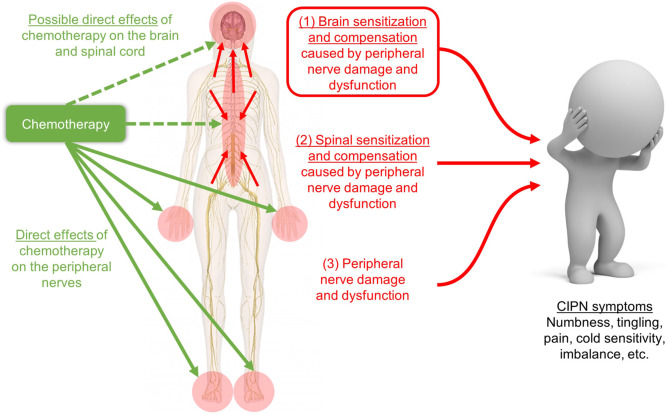
Schematic of our hypothesis that CIPN symptoms are caused by (1) brain sensitization and compensation due to peripheral and spinal nerve damage and dysfunction, which is shown in the red box and is the focus of our novel review, plus two more well-studied phenomena: (2) spinal sensitization and compensation, and (3) peripheral nerve damage. Our hypothesis does not depend on whether chemotherapy enters the brain (green dashed arrows) for changes in the brain to contribute to CIPN symptoms. Image adapted from innerbody.com.

We hypothesize that the central nervous system (CNS), and particularly the brain, has a previously under-recognized role in the pathophysiology of human CIPN (Figure 1). This novel perspective can dramatically shift our understanding of CIPN, inform new avenues of research, and ultimately accelerate the development of new and more effective clinical methods to diagnose, treat, and prevent CIPN. Our perspective is consistent with the fact that the only proven treatment for CIPN (duloxetine) acts in the brain as a serotonin-norepinephrine reuptake inhibitor (Smith and Nicholson, 2007; Smith, Pang et al., 2013). The importance of the brain is also emphasized by the well-known poor correlations between peripheral nerve conduction results and patient symptoms of CIPN (Cavaletti et al., 2011; Sharma et al., 2015). This apparent discrepancy is actually consistent with a massive body of literature from psychology and neuroscience that human feelings (including symptoms) are not flawless reflections of peripheral sensory input but instead feelings are a loose interpretation or prediction merely *tailored* by peripheral sensory input (Kleckner and Quigley, 2015; Siegel et al., 2018; Barrett and Satpute, 2019). In addition, the brain may play a role in CIPN even if neurotoxic chemotherapy does not enter the brain; indeed, the brain undergoes compensation and reorganization due to peripheral damage in other conditions such as phantom limb pain (Makin and Flor, 2020). Several studies have hypothesized or studied brain mechanisms in CIPN in humans (Weng et al., 2003; Boland et al., 2014; Dougherty, 2016; Nudelman et al., 2016; Prinsloo et al., 2017; Kleckner et al., 2018), and in rodents (e.g. (Thibault et al., 2012; Ferris et al., 2019)). Yet, to date, no papers have synthesized the current state of knowledge regarding the role of the brain in CIPN.

The goal of this review is to begin to answer two questions that have not been comprehensively addressed in the literature: (1) *does the brain play a prominent or even causal role in the pathophysiology of CIPN (i.e., CIPN as a syndrome, not just the peripheral neuropathy itself)*? and (2) *how can we leverage knowledge of the brain’s role in CIPN to accelerate basic research, clinical research, and diagnostics, treatment, and prevention of CIPN*? We performed a scoping review (Munn et al., 2018) to synthesize evidence from published studies in humans that assessed relationships between CIPN severity and brain measures (e.g., activity, blood flow) and studies in non-human animals that used experimental manipulations of the brain and observed its effects on CIPN. We synthesized the results of these papers at the molecular and brain network/systems level. Finally, we consider implications for preclinical research, clinical research, and clinical treatment of CIPN informed by the proposed mechanisms of brain involvement.

## Methods

We conducted a literature review on brain interventions in CIPN using PubMed. Our criteria were as follows: each study (1) measured CIPN signs or symptoms, (2) included human or non-human primate imaging OR an intervention delivered to the brain or spinal cord, (3) was written in English, and (4) was published before January 2021. We began with two PubMed searches: (1) (oxaliplatin OR paclitaxel OR docetaxel OR cisplatin OR bortezomib OR thalidomide) AND (insula OR insular OR cingulate OR cortex OR cortical OR amygdala OR somatosensory OR thalamus OR brain) NOT kidney NOT renal NOT nephrotoxicity, and (2) (ICV or intracerebroventricularly) AND neuropathy AND (oxaliplatin OR carboplatin OR paclitaxel OR docetaxel), and then we identified additional papers of interest by searching papers that cited the papers from our PubMed search.

We conducted exhaustive searches of the literature on the role of the human/primate brain in CIPN and of brain interventions in CIPN. However, we did not conduct an exhaustive search of all spinal cord intervention papers; rather, we selected papers that also mentioned the role of the brain, per our literature search criteria.

## Results and Discussion

### Human and Non-human Primate Brain Imaging (5 Studies; Table 2)

Our literature search found three published studies of the brain and CIPN in humans, and two studies in macaque monkeys (details provided in Table 2). The human studies were fairly heterogeneous in terms of patients (breast, multiple myeloma, mixed cancers), design (comparing pre- and post-chemotherapy, case-control of patients with vs. without CIPN, RCT using EEG neurofeedback), and sample size (range of 7–62). Three studies used fMRI in response to an applied painful stimulus, one study assessed resting blood perfusion with MRI, and one study assessed resting power in various frequency bands using EEG.

**TABLE 2:** Human and non-human primate studies of the brain and CIPN.

Citation	Sample, size, design	Type of chemotherapy	CIPN measures and results	Brain measures and results
Nudelman et al. (2016)	*47 women with non-metastatic breast cancer*	Various combinations of paclitaxel, docetaxel, carboplatin, and cisplatin across patients as part of adjuvant (N = 16) or neoadjuvant (N = 8) standard-dose chemotherapy regimens	*Method*	*Method*
	24 Treated with chemotherapy		Patient-reported functional assessment of cancer Therapy/Gynecologic Oncology Group–Neurotoxicity four-item sensory-specific scale	–All brain measures occurred in chemotherapy patients only
	23 Without chemotherapy		*Results*	–3T MRI scanner wtih 12-channel head coil
	*Assessed 3 times*		CIPN symptoms were more severe at 1 month and 13 months post-chemotherapy compared to cancer controls at matched time intervals	–Arterial spin labeling (ASL) MRI to assess perfusion at rest (eyes closed)
				–structural MRI to assess gray matter density
	Before treatment			*Results*
	1 month after treatment completion			–At 1 month CIPN severity was associated with greater perfusion in the superior frontal gyrus, cingulate gyrus, left middle gyrus, medial frontal gyrus
	1 year after the 1-month assessment			–Increase in CIPN severity from pre- to 1-month-post chemotherapy was associated with greater perfusion in the left cingulate gyrus and left superior frontal gyrus
				–At 1 year, no significant associations between CIPN severity and brain perfusion
				–Decreased gray matter density in left middle/superior frontal gyrus from pre- to 1-month-post chemotherapy was associated with decreases in both CIPN severity and perfusion
Boland et al. (2014)	*24 Individuals*	After receiving bortezomib, thalidomide, or vincristine	*Method*	*Method*
	12 With multiple myeloma and CIPN		Total neuropathy score (TNS), reduced (clinical analysis of motor and sensory signs and symptoms)	–Brain fMRI reactivity to noxious heat-pain stimulation on the right foot and thigh (7/10 pain rating) vs. warm stimulation (32°C)
	12 Healthy volunteers	Reporting neuropathic pain for at least 6 months (range 0.9–3.2 years, median 2 years)		
				*Results*
	Assessed once			–Patients exhibited greater activation in the left precuneus, and lower activation in the right superior frontal gyrus for both foot and thigh compared to healthy volunteers
				–Activation in the left frontal operculum (near the insula) in response to heat-pain stimulation of the foot was associated with worse CIPN
Prinsloo et al. (2017)	*62 Cancer survivors with CIPN (mostly breast)*	Various combinations of taxane and platinum agents	*Method*	*Method*
	30 Randomized to neurofeedback		–Patient-reported brief pain inventory (BPI)	–EEG recording using 19-electrode cap for 10 min eyes open, 10 min eyes closed
	32 Randomized to waitlist control	Reporting CIPN for at least 3 months after completing chemotherapy	–Pain quality assessment scale (PQAS)	–Neurofeedback was designed to increase power in the alpha band (8–12 Hz)
				–LORETA to localize EEG results to a brain map
	*Assessed 2 times*		*Results*	*Results*
	Pre-intervention		–Neurofeedback reduced worst pain, average pain, and features of pain (e.g., unpleasantness) compared to waitlist control	–Neurofeedback increased alpha power and decreased beta power compared to control
	Post-intervention (after 20 sessions, up to 10 weeks)			–Decrease in beta power was correlated with decrease in worst pain in bilateral parietal, frontal, central, and parietal midline regions
				–No associations between increase in alpha power or alpha/beta ratio and worst pain
				–Neurofeedback increased activity in the dorsolateral prefrontal cortex and decreased activity in the insula, with no differences in the rostral ACC compared to control
Shidahara et al. (2019)	8 Macaque monkeys	Oxaliplatin (5 mg/kg) infused intravenously over 2 h, then again 3 weeks later	–A prior study by this group showed that duloxetine was anti-nociceptive whereas pregabalin and tramadol were not (Shidahara, Ogawa et al., 2016)	*Method*
	–4 Received vehicle or tramadol first (in infusion 1)	Assessments performed 3 days after oxaliplatin infusion		–Brain MRI scan performed 3 days after oxaliplatin infusion
	–4 Received pregabalin or duloxetine first (in infusion 1)			–Blocks of 30 s of cold stimulation (10°C) vs. 30 s of warm stimulation (37°C) to the tail
				*Results*
				–After oxaliplatin, the S2 and insula exhibited greater activity in response to cold stimulation to the tail (compared to pre-oxaliplatin)
				–Duloxetine reduced S2 and insula activation in response to cold stimulation, whereas pregabalin and tramadol did not
Nagasaka et al. (2017)	Male adult cynomolgus macaque monkeys (*Macaca fascicularis*)	–Oxaliplatin	–Oxaliplatin (post vs. pre) decreased withdrawal latency to cold stimulation to the tail (allodynia)	–Oxaliplatin (post vs. pre) enhanced brain activity in S2/insula in response to cold stimulation to the tail
	7 Total—all oxaliplatin treated	–5 mg/kg intravenous injection over 2 h		–Duloxetine reduced S2 and insula activation in response to cold stimulation
	–4 fMRI (pre vs. post-oxaliplatin)	–fMRI conducted 3 days after oxaliplatin injection		
	–2 vs. 1 muscimol vs. vehicle microinjection to secondary somatosensory cortex (S2) and insula			

We identified three common themes across these five papers. First, CIPN is associated with brain hyperactivity in response to painful stimuli in sensory regions (S2/insula; Boland et al., 2014; Shidahara et al., 2019; and Nagasaka et al., 2017)^1^ and in the posterior portion of the default mode network (DMN (Raichle, 2015); specifically, the ventral precuneus; Boland et al., 2014) but reduced activity in anterior DMN (superior frontal gyrus, strongly connected to the ACC and mid cingulate cortex (Li et al., 2013; Boland et al., 2014). Second, CIPN is associated with greater resting perfusion in the DMN (superior frontal gyrus, cingulate; medial frontal gyrus; Nudelman et al., 2016) and greater gray matter densities in the same regions (Nudelman et al., 2016). Third, in terms of interventions, reduction of CIPN symptoms is associated with reduction in brain activity in the insula (Shidahara et al., 2019; Nagasaka et al., 2017; and Prinsloo et al., 2017). Duloxetine reduced the brain response to cold-induced pain in S2/insula (Shidahara et al., 2019 and Nagasaka et al., 2017), and reduction in CIPN pain from neurofeedback was associated with a reduction in insula activity at rest (Prinsloo et al., 2017) as well as a reduction in resting β power (13–45 Hz) in the bilateral parietal cortices and midline regions (including the ACC and DMN; Prinsloo et al., 2017).

The aforementioned studies suggest that hyperactivity in the brain (particularly the insula) is positively correlated with CIPN severity. However, because of the observational nature of these studies,^2^ it is unclear whether brain changes causally contribute to changes in CIPN symptoms, or whether the brain changes are merely epiphenomenal. To explore how changes in the brain might cause changes in CIPN symptoms, we next reviewed studies in non-human animals testing interventions to the brain itself, with results supported by interventions to the spinal cord.

### Overview of Rodent Studies With CNS Interventions (24 Studies)

Table 3 shows all 16 studies using brain interventions from our literature search. Six studies used injections of specific receptor agonists or antagonists or other compounds applied to a localized brain region, nine used intracerebroventricular (ICV) injections, and one used *ex-vivo* slices from a specific brain region. All studies in Table 3 used either oxaliplatin or paclitaxel. Most used mice or rats as their animal model (often male), while one used monkeys. The brain regions investigated include the periaqueductal gray (PAG), thalamus, anterior cingulate cortex (ACC), insula, secondary somatosensory cortex (S2), and sometimes less specifically defined regions such as “frontal cortex” (likely including the ACC) or the entire cortex. Brain measures included fMRI, synaptic potentials, and PCR and western blot analysis on post-mortem brain sample homogenates.

**TABLE 3:** Studies that test interventions to the brain that cause or treat CIPN symptoms.

Citation	Sample size and study design	Chemotherapy regimen	Effect of chemotherapy on CIPN symptoms and brain	Brain intervention and its effects on CIPN symptoms and brain	Conclusion
Costa et al. (2011)	Mice	–Paclitaxel 2 mg/kg intraperitoneally for 5 consecutive days	–Paclitaxel decreased mechanical and thermal threshold in wild type C57 and CD1 mice	–*Intervention*: DALBK (selective kinin B_1_ R antagonist) and Hoe 140 (selective kinin B_2_ receptor antagonist) administered to wildtype mice intraperitoneally (systemic), intraplantary (peripheral), intrathecally (spinal), or ICV (central)	–Paclitaxel induced mechanical and thermal hypersensitivity in wildtype mice
	30 mice total—all paclitaxel treated			–Systemic treatment with DALBK or Hoe 140 inhibited the mechanical and thermal hyperalgesia induced by paclitaxel	–Paclitaxel treatment increased expression of the B_1_ receptor transcript in the thalamus and PFC, but reduced their basal expression in the hypothalamus
	–6 adult CD1 wild-type mice vs. vehicle controls	–Pain sensitivity tests began on day 7 from the first paclitaxel administration, until day 14 or 21	–Paclitaxel-treated kinin B_1_ or B_2_ receptor- knockout mice exhibited a lower frequency of response to both mechanical and thermal stimuli vs. wildtype mice		–Knocking out of either the kinin B_1_ or B_2_ receptors decreased the paclitaxel-induced hyperalgesia. Knocking out both receptors further decreased the hyperalgesia
	–6 Male C57BL/G wild-type mice		–Inhibition of paclitaxel-induced hyperalgesia by the B_1_B_2_R^−/−^ double knock-out mice was greater than that caused by single ablation of B_1_ or B_2_ receptors	–Peripheral treatment with DALBK or Hoe 140 did not alter the paclitaxel-induced mechanical hyperalgesia	
	–6 C57BL/6 kinin B1 R-knockout mice				–Systemic and central, but not peripheral treatment with B_1_ or B_2_ receptor antagonists inhibited the mechanical and thermal hyperalgesia, suggesting that kinin rs do not contribute to paclitaxel-induced mechanical hyperalgesia at the peripheral level
	–6 C57BL/6 kinin B2 R- knockout mice		–5 Days treatment with a single paclitaxel injections induced an over-expression of kinin B1 receptor transcripts in the mouse thalamus and pre-frontal cortex (PFC)	–Intrathecal treatment with DALBK or Hoe 140 significantly inhibited mechanical hyperalgesia	
	–6 Mice lacking the genes encoding both kinin receptors (double knockout)				
			–Paclitaxel administration reduced the basal level of kinin B_1_ receptor expression in the mouse hypothalamus	–ICV treatment with DALBK or Hoe 140 did not alter paclitaxel-induced mechanical hyperalgesia when administered on the seventh day	
				–A second ICV treatment to the same group 14 days following the first paclitaxel treatment inhibited mechanical hyperalgesia with DALBK but not Hoe 140	
Ferrier et al. (2015)	Male Sprague Dawley rats 213 rats total	–Oxaliplatin 2 mg/kg intravenously twice/week for 4.5 weeks	–Oxaliplatin increased withdrawal to electronic von Frey (mechanical allodynia) –Oxaliplatin decreased acetylcholine (Ach) in the posterior insula, increased choline in the posterior insula, and decreased GABA in the thalamus –Oxaliplatin increased transcript expression of cholinergic receptors (Chrm2, Chrnb4, Chrna7) and choline transporter (Slc5a7; CHT1) in the posterior insula –Oxaliplatin increased M2R protein expression in posterior insula	–*Intervention*: Oxotremorine (muscarinic R agonist), Methoctramine (selective M2R antagonist) and Donepezil (reversible acetylcholinesterase inhibitor) injections into the posterior insula. Also, systemic (oral) Donepezil administration	–Oxaliplatin caused metabolic changes in the insula and thalamus, including an increase in choline and a decrease in GABA, as well as an increase in M2R in the posterior insula –Injecting M2R agonist into the posterior insula reversed CIPN symptoms –Injecting an AChE inhibitor increased levels of ACh in the posterior insula, and systemic AChE inhibitor reduced CIPN symptoms
				–Oxotremorine injected in the posterior insula reduced mechanical allodynia, and had no effect on oxaliplatin-naïve rats	

				–Methoctramine injected into the posterior insula prevented anti-allodynic effects of Oxotremorine, and had no effect on its own	

				–Systemic Donepezil reversed mechanical and cold allodynia and decreased fall latencies	

				–Systemic Donepezil taken before oxaliplatin prevented CIPN symptoms	

				–Donepezil injection into the posterior insula increased ACh levels	
Hache et al. (2015)	Male C57BL6j mice	–Oxaliplatin (7 mg/kg) intraperitoneally daily for 2 days, followed by 2 days of rest, then 2 days of injection, then 2 days of rest, then assessments (4 injections total)	–Oxaliplatin increased paw withdrawal frequency in the von Frey test in comparison vehicle injected mice, causing mechanical hypersensitivity –Oxaliplatin treatment induced cold allodynia and hyperalgesia	–*Intervention*: Each of several agents delivered to the anterior cingulate cortex (ACC) via micro dialysis –NS18283 triple monoamine reuptake inhibitor (serotonin, norepinephrine, dopamine) –INDATRALINE triple monoamine reuptake inhibitor (serotonin, norepinephrine, and dopamine) –Venlafaxine selective norepinephrine reuptake inhibitor (serotonin and norepinephrine) –Escitalopram selective serotonin reuptake inhibitor (serotonin) –Each reuptake inhibitor increased levels of its respective monoamine(s) in the ACC –Indatraline reversed all CIPN symptoms –NS18283 reversed mechano-hypersensitivity and cold allodynia –Venlafaxine reversed only cold allodynia –Escitalopram reversed only mechano-hypersensitivity	–Oxaliplatin induced mechanical hypersensitivity, cold allodynia, and cold hyperalgesia –Reuptake inhibitors of serotonin, norepinephrine, and dopamine each delivered to the ACC reversed different components of the oxaliplatin-induced CIPN symptoms
Juarez-Salinas et al. (2018)	C57BL6 mice 18 total mice –8 Received paclitaxel –10 Received vehicle	–Paclitaxel 1 mg/kg intraperitonially every other day for 4 total injections –Assessments one week after final paclitaxel injection	–Paclitaxel induced mechanical hypersensitivity	–*Intervention*: ICV injection of gabapentin (GP, voltage-gated Ca^2+^ channel inhibitor) into the left lateral ventricle. Simultaneous GP 100 μg ICV injection + Yohimbine (α_2_ receptor antagonist) intrathecal injection –100 μg ICV GP in paclitaxel-treated mice showed reduced mechanical allodynia and increase in place preference for the GP-paired side of the apparatus –Supraspinal GP administered at a dose that does not reverse mechanical allodynia (30 μg) did not relieve pain in paclitaxel-treated mice –Simultaneous GP and Yohimbine injection eliminated the preference for the gabapentin-paired chamber	–Paclitaxel induced mechanical hypersensitivity –Gabapentin injection to the brain (ICV) reduced both mechanical hypersensitivity and pain aversiveness in a dose-dependent manner
Kanat et al. (2013)	Male Sprague Dawley rats	–Oxaliplatin 6 mg/kg single dose intraperitoneally –Experiments performed on the second day following oxaliplatin treatment	–Oxaliplatin decreased the paw withdrawal threshold in response to mechanical pressure	–*Intervention*: 0.5, 1.0, and 2.0 μmol CDP-choline (increases tissue choline and ACh) delivered intracerebroventricularly (ICV) –CDP-choline reduced mechanical hyperalgesia in a dose- and time-dependent manner –Effects of CDP-choline were blocked by ICV delivery of –Choline uptake inhibitor hemicholinium-3 –nonselective nicotinic receptor antagonist mecamylamine –α7 selective nicotinic acetylcholine receptor antagonist α-bungarotoxin –GABA_B_ receptor antagonist CGP-35348 –Effects of CDP-choline were *not* blocked by ICV delivery of –Nonselective opioid receptor antagonist naloxone –Nonselective muscarinic receptor antagonist atropine	–Oxaliplatin induced mechanical hyperalgesia –CDP-choline delivered to the brain (ICV) reduced CIPN symptoms (mechanical hyperalgesia) in a manner dependent upon choline uptake, nicotinic receptor activity, and GABA receptor activity but not opioid or muscarinic receptor activity
Kanbara et al. (2014a)	Male Sprague Dawley rats	–Oxaliplatin 2 mg/kg or 4 mg/kg intraperitoneally, twice/week for 4 weeks –Paw withdrawal thresholds were assessed before oxaliplatin treatment, and on days 1, 8, 15, 22, 29 and 36 following treatment	–Oxaliplatin resulted in a dose-dependent decrease in weight gain in comparison to control –Oxaliplatin resulted in decreased paw withdrawal thresholds in comparison to control –Oxaliplatin resulted in decreased mean and peak sciatic nerve conduction velocity in comparison to control	–*Intervention*: ICV Pertussis toxin (PTX; a selective Gi/o protein inhibitor) –Anti-nociceptive effects of morphine and oxycodone but not fentanyl (each delivered subcutaneously) were blocked by PTX delivered to the brain –Oxaliplatin reduced drug-induced activation of the μ-opioid receptor in the thalamus for fentanyl but not for morphine and oxycodine (no oxaliplatin vs. control differences in the PAG or spinal cord)	–Oxaliplatin caused mechanical hypersensitivity and decreased nerve conduction velocity –PTX-sensitive G-protein in the brain mediated the antinociceptive effects of morphine and oxycodone, but not fentanyl
Kanbara et al. (2014b)	Male Sprague Dawley rats	–Oxaliplatin 2 mg/kg intraperitoneally, twice/week for 4 weeks	–Oxaliplatin resulted in decreased paw withdrawal thresholds in comparison to control (as per previous/above study)	–*Intervention*: 30 pmol ICV and intrathecal Tertiapin-Q (a GIRK1 channel blocker) –ICV Tertiapin-Q blocked anti-nociception of oxycodone, but not morphine nor fentanyl –Intrathecal (spinal cord) Tertiapin-Q blocked anti-nociception of morphine, but not oxycodone nor fentanyl	–GIRK1 channels mediate the anti-nociceptive effects of morphine and oxycodone at different levels in the neuraxis (oxycodone via brain GIRK1 channels, morphine via spinal GIRK1 channels, fentanyl via neither)
Nagasaka et al. (2017)	Male adult cynomolgus macaque monkeys (*Macaca fascicularis*) 7 total (all received oxaliplatin) –4 fMRI (pre vs. post-oxaliplatin) –2 vs. 1 muscimol vs. vehicle microinjection to secondary somatosensory cortex (S2) and insula (ins)	–Oxaliplatin 5 mg/kg intravenously over 2 h –fMRI conducted 3 days after oxaliplatin injection	–Oxaliplatin (post vs. pre) decreased withdrawal latency to cold stimulation to the tail (allodynia) –Oxaliplatin (post vs. pre) enhanced brain activity in S2/Ins in response to cold stimulation to the tail	–*Intervention*: Muscimol (GABA_A_ receptor agonist) vs. vehicle injection into S2/insula. Duloxetine (selective serotonin and norepinephrine reuptake inhibitor) systemic injection without vehicle/control injection –Muscimol increased withdrawal latency to cold stimulation –Duloxetine increased withdrawal latency and prevented cold-induced activation of S2/Ins	–Oxaliplatin caused hyperexcitability of S2/insula during cold stimulation –Experimentally activating the GABA pathway (increasing neural inhibition) via direct injection to S2/insula reversed CIPN symptoms
Nashawi et al. (2016)	Male Sprague Dawley rats 108 –43 Control –21 Vehicle-treated –44 paclitaxel-treated	–Paclitaxel 2.67 ml/kg intraperitoneally on 2 alternate days –Measures performed 7 days after paclitaxel	–Paclitaxel reduced withdrawal threshold to mechanical stimuli –Paclitaxel caused stronger excitatory synaptic strength signal (higher E_max_) in the ACC	–*Intervention*s: GABA, E139 (an anticonvulsant that enhanced extracellular GABA levels) and CGP (GABA_B_ antagonist) bath applications to the ACC (post-mortem) –GABA reduced ACC field excitatory post synaptic potential (fEPSP) slope and restored their excitability levels to those of untreated mice ACCs –E139 reduced ACC fEPSP slopes in paclitaxel-treated mice –CGP increased ACC E_max_ in paclitaxel naive rats, but had no effect on paclitaxel-treated rats	–Paclitaxel induced mechanical hypersensitivity and hyperexcitability in the ACC –Restoration of GABA levels through direct GABA application or E139 decreased hyperexcitability in ACC slices –Antagonizing GABA increased excitability in ACC slices of paclitaxel-naïve rats
Norcini et al. (2009)	Male Sprague Dawley rats	–Oxaliplatin 2.4 mg/kg intraperitoneally 5 days/week for 3 weeks (chronic oxaliplatin)	–Oxaliplatin reduced paw withdrawal threshold and mechanical nociceptive threshold –At day 21, oxaliplatin increased PKCγ (but not PKCε) in the thalamus and PAG –Oxaliplatin increased phosphorylated PKCγ and PCKε isoforms in the thalamus and PAG, and PKCγ in the striatum, but neither in the spinal cord –Oxaliplatin increased phosphorylated p38MAPK level in the PAG and thalamus (no significant change in p38MAPK protein levels) –Oxaliplatin increased phosphorylated ERK1/2 levels in cortex and spinal cord, and decreased them in the striatum, thalamus and PAG –Oxaliplatin increased phosphorylated SAPK/JNK levels in striatum and cortex, and decreased them in the thalamus –Oxaliplatin increased *p*-ERK1/2 levels in cortex and spinal cord, and decreased them in the striatum, thalamus, and PAG –Oxaliplatin increased *p*-SAPK/JNK levels in striatum and cortex, and decreased them in the thalamus	–*Intervention*: 5 uL ICV Calphostin C (PKC inhibitor) injection to the left lateral ventricle –ICV administration of Calphostin C acutely (within 1–2 h) reversed mechanical hyperalgesia in a dose-dependent manner –Calphostin C resulted in a complete reversal of PKCγ phosphorylation in the thalamus, and a partial reversal in the PAG, with no changes in the spinal cord –Calphostin C restored basal phosphorylation levels of PKCε in the thalamus and PAG –Calphostin C reversed phosphorylated p38MAPK values to control in the thalamus and PAG	–Chronic oxaliplatin (21 days) increased phosphorylation of PKC and other downstream second messengers (e.g., MAPK, JNK) in the thalamus and PAG –Experimentally inhibiting PKC in the brain (ICV) reduced symptoms of CIPN (pressure hyperalgesia) within 1–2 h and partially normalized phosphorylation of PKC and MAPK
Sanna et al. (2016)	Male CD1 mice –10-15 oxaliplatin treated vs. 10 vehicle controls –10–15 Calphostin C ICV injection 21 days after oxaliplatin administration	–Oxaliplatin 2.4 mg/kg intraperitoneally 5 days/week for 3 weeks –Experiments carried out on days 14, 21 and 28	–Oxaliplatin reduced thermal nociceptive threshold to hot plate test at 4 different temperatures –Oxaliplatin reduced phosphorylated neurofulament H (pNfH; for the cytoskeleton) expression in the SC on day 21 and thalamus on day 28 but increased in the spinal cord and cortex on day 28. There was no effect at the PAG –Oxaliplatin reduced growth-associated protein-43 (GAP43; axonal growth) in the thalamus and PAG on day 28 –Oxaliplatin decreased HuD (RNA-binding protein associated with GAP43) in the spinal cord and cortex on day 28 –Oxaliplatin increased phosphorylated PKCγ in the thalamus and PAG	–*Intervention*: 5 uL ICV (unspecific location) Calphostin C –ICV administration of Calphostin C completely prevented the oxaliplatin-induced decrease of pain threshold	–Oxaliplatin reduced levels of proteins involved in neural outgrowth, synaptogenesis and maintenance of normal morphology, until this pattern reversed with compensatory neurogenesis seen by day 28 post-oxaliplatin –Oxaliplatin increased levels of PKCγ in the thalamus and PAG –Experimentally inhibiting PKC in the brain (ICV) completely reversed symptoms of CIPN (thermal hyperalgesia)
Stine et al. (2020)	Young adult male and female CD-1and female BALB/cfC3H mice	–Paclitaxel 2 mg/kg intraperitoneally on days 1,3,5, and 7	–Paclitaxel reduced mechanical allodynia threshold	–*Intervention*: Heat shock protein 90 (Hsp90) inhibitors given ICV, intrathecally or intraperitoneally –Hsp90 inhibitors given ICV or intraperitoneally blocked morphine anti-nociception in CIPN	–Paclitaxel caused mechanical allodynia –Hsp90 inhibitors delivered to the brain (ICV) interfered with opioid pain management for CIPN
Thibault et al. (2012)	Male Sprague Dawley rats 55 total –19 oxaliplatin treated vs. 18 vehicle controls –10 shRNA lentiviral vectors (silencing Kcnb2 mRNA) vs. 8 vehicle control injections to left and right hind limb somatosensory cortex	–Oxaliplatin 4 mg/kg intraperitoneally, twice/week for 4.5 consecutive weeks	–Oxaliplatin increased responses to smooth and rough paintbrush tests (allodynia) and decreased response to electronic von Frey and pinch tests (hyperalgesia) –Oxaliplatin downregulated genes in somatosensory cortex related to signal transduction, cell metabolism, transcription regulation, RNA polymerase II, and the Kv2.2 voltage-dependent K^+^ channel –Oxaliplatin increased number of *p*-Erk-IR neurons (marker for neuronal activity) in the primary somatosensory, cingulate, and motor cortices	–*Intervention*: Downregulation of Kv2.2 in chemotherapy-naïve rats using injection of shRNA lentiviral vector in the somatosensory cortex, which caused –Sustained cold and mechanical hypersensitivity –Decreased responses to electronic von Frey –Increased awareness and nociceptive threshold in cold plate test –Increased number of neurons immunoreactive for *p*-Erk-IR	–Oxaliplatin increased activity in the somatosensory cortex –Oxaliplatin downregulated nearly all genes in the somatosensory cortex, including genes for K^+^ channels –Experimentally down-regulating K^+^ channel expression in the somatosensory cortex increased neural activity and caused sensory symptoms of CIPN
Toyama et al. (2017)	Male BALB/c mice	–Oxaliplatin 10 mg/kg intraperitoneally, once/week for 3 weeks (days 1, 8, and 15)	–Oxaliplatin induced mechanical allodynia –Oxaliplatin induced acute thermal pain	–*Intervention*: ICV Orexin-A (neuropeptide) delivery to the lateral ventricle –Systemic (intraperitoneal) delivery of SB-408124 (orexin type-1 receptor antagonist) and TCS-OX2-29 (orexin type-2 receptor antagonist) (both compounds can cross the blood-brain barrier) –ICV Orexin-A reduced mechanical allodynia and thermal pain in a dose-dependent manner –Effects of orexin-A were blocked by systemic SB-408124 but not by TCS-OX2-29	–Oxaliplatin induced mechanical allodynia and thermal hypersensitivity –Orexin-A delivered to the brain (ICV) reduced CIPN symptoms, and these effects were blocked by an orexin type-1 receptor antagonist, but not a type-2 receptor antagonist
Xu et al. (2018)	Male Sprague Dawley rats 12 rats –20 oxaliplatin-treated vs. 18 controls	–Oxaliplatin 6 mg/kg intraperitoneally –Experiments performed 3 days after injection	–Oxaliplatin caused mechanical and cold hypersensitivity –Oxaliplatin increased levels of IL-1β, IL-6, TNF-α, and pro-inflammatory cytokine receptors in the dorsolateral periaqueductal gray (dl-PAG) –Oxaliplatin increased ratio of membrane and total PIC receptor densities in the dl-PAG –Oxaliplatin decreased levels of GABA in the dl-PAG	–*Intervention*: The following injections into the dl-PAG using a pump –IL-1Ra (IL-1β receptor antagonist) –SC144 (IL-6 R gp130 antagonist) –Etanercept (TNF-α receptor antagonist) –Muscimol (GABA_A_ receptor agonist) –Blocking pro-inflammatory cytokine receptors in the dl-PAG reduced mechanical and cold allodynia –Blocking pro-inflammatory cytokine receptors in the dl-PAG restored decreased GABA –Stimulating the GABA_A_ receptor through muscimol in the dl-PAG reduced mechanical and cold allodynia	–Oxaliplatin induced mechanical and cold hypersensitivity –Oxaliplatin increased levels of pro-inflammatory cytokines and their receptors, and decreased levels of GABA in the dl-PAG –Blocking pro-inflammatory cytokine receptors in the dl-PAG (direct injection) alleviated CIPN symptoms and restored GABA levels –Activating GABA_A_ receptors in the dl-PAG alleviated CIPN symptoms
Zhang et al. (2019)	Male Sprague Dawley rats 70 total	–Paclitaxel 1 mg/kg on days 0, 2, 4, 6	–Paclitaxel caused a 40–60% reduction in mechanical threshold compared to day 0 of paclitaxel treatment –Paclitaxel caused a cold allodynia	–*Intervention* –Hyperbaric oxygen (HBO_2_) treatment; animals were placed in a hyperbaric chamber ventilated with 100% O_2_ for 60 min –S-Methyl_l-thiocitruilline (SMTC, a neuronal nitric oxide synthase (nNOS) inhibitor) delivered to the lateral ventricle (ICV) –HBO_2_ treatment alleviated mechanical allodynia (after 1 treatment) and cold allodynia (after 4 daily treatments) –HBO_2_ treated rats had significantly higher mechanical and cold allodynia thresholds than rats not receiving HBO_2_ –The benefit of HBO_2_ on allodynia was reduced by lateral ventricle infusion of SMTC	–Paclitaxel caused mechanical and cold allodynia –Hyperbaric treatment reduces CIPN symptoms, but that is blocked via blocking NO synthase in the brain (ICV)

Table 4 includes the eight papers using interventions to the spinal cord via intrathecal injection or cell transplant. In comparison to studies of brain interventions, the spinal cord intervention papers utilized a wider variety of chemotherapy agents including oxaliplatin, paclitaxel, and vincristine. Additionally, Table 4 papers focused on more detailed, molecular-level analyses of CNS changes. Across all studies, CIPN signs and symptoms were evaluated using various tests including the Electronic von Frey, tail immersion test, hot and cold plate tests, paw pressure tests, and rotarod tests. Table 4 provides in depth details regarding the methods and outcomes of these studies, while the below results section provides a broader summary of the main results.

**TABLE 4:** Papers that test interventions to the spinal cord that cause or treat CIPN symptoms.

Author	Sample size and study design	Chemotherapy regimen	Effect of chemotherapy on CIPN symptoms and brain	Effects of CIPN intervention on the brain	Conclusion
Bráz et al. (2015)	C57BL/6 male mice VGAT mutant mice (deletion of vesicular GABA transporter)	–Paclitaxel 1 mg/kg intraperitoneally 4 times every other day –Transplantation 1 week after hypersensitivity development	–Paclitaxel caused mechanical and heat hypersensitivity –Paclitaxel decreased spinal cord expression of glutamic acid decarboxylase (GAD65 and 67; enzymes catalyzing the conversion of glutamate to GABA) –number of ATF3-positive (marker of sensory neuron damage) DRG neurons did not differ in the spinal cord of the paclitaxel and vehicle mice, and was lower than the peripheral nerve injury models –Levels of Iba-1 (marker of activated microglia) expression did not differ in the spinal cord of the paclitaxel and control mice, and the peripheral nerve injury produced a much greater activation of microglia	–*Intervention*: Transplantation of MGE cells to restore GABAergic signaling in the spinal cord of wild-type and mutant mice –MGE transplantation in wild type mice reduced both mechanical and heat hypersensitivity; especially notable in heat –The transplant normalized GAD mRNA levels –MGE transplantation in VGAT mutant mice (lacking GABA transporter) did not reverse the mechanical or heat hypersensitivity	–Paclitaxel produced mechanical and heat hypersensitivity and decreased spinal expression of GABA-producing enzymes –Injection of MGE cells that release GABA in the spinal cord mediated the reversal of the mechanical and heat hypersensitivities –MGE of mice with deletion of the vesicular GABA transporter (VGAT mutant) gene did not reverse hyperalgesia, suggesting that GABA itself caused the reduction in CIPN symptoms
Luo et al. (2019)	C57BL/6J mice	–Single paclitaxel 6 mg/kg intraperitoneal injection or multiple 2 mg/kg intraperitoneal injections on days 0, 2, 4, and 6	–Paclitaxel caused mechanical allodynia, increased IL-17 in the CSF and spinal cord dorsal horn –IL-17R mRNA expressed on SOM^+^ neurons in the spinal dorsal horn –More positive resting membrane potential and a lower rheobase were observed in somatostatin-expressing neurons (SOM^+^; excitatory interneurons) neurons –Greater number of action potential firings in small-sized DRG neurons	–*Interventions* –IL-17 intrathecally –IL-17R-shRNA injected in the intra-dorsal horn of SOM-Cre mice –GABA and Glycine bath application –IL-17 caused a transient reduction of paw withdrawal threshold and increased the amplitude of NMDAR-EPSC evoked by dorsal root entry zone –SOM^+^ perfusion with IL-17 induced a rapid increase in the frequency but not amplitude of sEPSCs –IL-17 inhibited GABA-induced currents but had no effect on glycine-induced currents in spinal SOM^+^ neurons –Blocking IL-17R with a neutralizing antibody resulted in opposite changes in excitatory and inhibitory synaptic transmission in lamina IIo SOM^+^ neurons of paclitaxel-treated animals –In DRG neurons, IL-17RA antibody treatment suppressed excitability increase –Knockdown of IL-17R in spinal SOM^+^ neurons delayed and suppressed paclitaxel-induced mechanical allodynia –Selective knockdown of IL-17R in spinal SOM^+^ neurons suppressed the frequency, but not the amplitude of sEPSCs	–Paclitaxel increased levels of pro-inflammatory cytokine IL-17, created a more positive resting potential in excitatory interneurons and increased neural activity –IL-17 enhanced excitatory synaptic transmission, potentiated NMDA-mediated eEPSCs in spinal cord slices, decreased the inhibitory control of SOM^+^ neurons and suppressed GABA-induced currents –IL-17 decreased inhibitory postsynaptic potentials and GABA-induced currents –Knockdown or blockage of IL-17 attenuated neural excitability and reversed CIPN symptoms
Mannelli et al. (2015)	Male Sprague Dawley rats –8 Rats/treatment in 2 different experimental sets	–Oxaliplatin 2.4 mg/kg intraperitoneally 5 days/week for 2 weeks –Cerebral cortex synaptosomes (purified nerve terminals) prepared on day 15 of oxaliplatin treatment	–Oxaliplatin induced mechanical hypersensitivity –Increased P2X7-evoked glutamate release from cerebrocortical synaptosomes –Higher ATP overflow in oxaliplatin-treated synaptosomes	–*Intervention*: BBG and A-438079 (P2X7 receptor antagonists) and Erioglaucine and^10^Panx (Pannexin 1 selective inhibitors) intrathecal *in-vivo* injections –P2X7-evoked glutamate release was eliminated by BBG and A-438079 –P2X7-evoked glutamate release was reduced by Carbenoxolone and Erioglaucine and^10^Panx –BBG, Erioglaucine and^10^Panx reversed oxaliplatin-induced pain	–Oxaliplatin induced mechanical hypersensitivity –Oxaliplatin increased P2X7R-depedant glutamate release in cerebrocortical nerve terminals, through Pannexin 1 recruitment –P2X7R antagonists and Pannexin 1 inhibitors eliminated or reduced the glutamate release, respectively, and eliminated oxaliplatin-induced pain
Morioka et al. (2019)	Male ddy mice	–Paclitaxel 2 mg/kg intraperitoneally once/day for 5 times every other day	–Paclitaxel caused mechanical hypersensitivity	–*Intervention*: intrathecal treatment of 100 or 300 nmol of SR9009 (agonist of REV-ERB, nuclear receptors related to regulation of metabolism, inflammation, and tumor growth) –SR9009 reduced the paclitaxel-induced mechanical hypersensitivity	–Paclitaxel induced mechanical hypersensitivity, which was significantly reduced by stimulating REV-ERB transcription factors
Maruta et al. (2019)	Male Sprague Dawley rats 5 rats	–Oxaliplatin 4 mg/kg intraperitoneally twice/week for 4 weeks –Electronic von Frey performed 1 week before and 1 week after oxaliplatin treatment	–Oxaliplatin caused mechanical allodynia –Increased ERK1/2 phosphorylation in the DRG up to 4.5-fold –Increased brain-derived neurotrophic factor (BDNF) in the DRG	–*Intervention*: PD98059 (ERK inhibitor) intrathecally –PD98059 inhibited mechanical allodynia –PD98059 inhibited upregulation of ERK phosphorylation in the DRG	–Oxaliplatin administration induced chronic mechanical allodynia and increased ERK1/2 phosphorylation in the DRG –ERK inhibitor prevented mechanical allodynia by inhibiting oxaliplatin-induced upregulation of ERK phosphorylation
Nie et al. (2018)	Male Sprague Dawley rats AKAP150^flox/flox^ mice (inhibition of AKAP150) –Control vehicle 12 rats in each group	–Paclitaxel 8 mg/kg intraperitoneally on 3 alternate days (days 1, 4 and 7, cumulative dose 24 mg/kg) in rats –Paclitaxel 2 mg/kg intraperitoneally for 5 consecutive days in mice	–Paclitaxel induced mechanical allodynia and thermal hyperalgesia –Paclitaxel increased mRNA and protein expression of A-kinase anchor protein 150 (AKAP150; accessory protein targeting enzymes involved in pain-related pathogenesis) in the DRG –Paclitaxel decreased enzyme activity of calcineurin (CN, a calcium and calmodulin dependent serine/threonine protein phosphatase that activates T cells) –Paclitaxel decreased nucleus NFAT2 (protein involved in T cell activation and differentiation) levels –Paclitaxel increased AKAP150 interaction with CN, decreased IL-10 mRNA (anti-inflammatory cytokine), decreased IL-13 mRNA (anti-inflammatory), which returned to normal level on day 10, decreased IL-4 mRNA (anti-inflammatory cytokine), and decreased NFAT2 binding to the IL-4 promoter in the DRG	–Interventions –AKAP150 siRNA (AKAP150 knockdown) –AKAP150flox/flox mice (AKAP150 inhibition) –Intrathecal FK506 (CN enzyme activity inhibitor) -AAV5-Cre-GFP (AKAP150 knockdown) –AAV5- NFAT2-GFP (overexpress NFAT2) –IL-4 siRNA (IL-4 knockdown) –Intrathecal CN –CN increased NFAT2 levels –AKAP150 siRNA attenuated the mechanical allodynia and thermal hyperalgesia –CN enzyme activity increased in AKAP150flox/flox mice injected with AAV5-Cre-GFP –AKAP150 knock down restored IL-4 –FK506 decreased NFAT2 expression in DRG nuclei –Intrathecal injection of IL-4 normalized hyperactivity of DRG neurons and attenuated mechanical allodynia and thermal hyperalgesia –NFAT2 increased after AAV5-NFAT2-GFP injections, which attenuated mechanical allodynia and thermal hyperalgesia –FK506 induced mechanical allodynia and thermal hyperalgesia –AAV5-NFAT2-GFP partly restored the decreased IL-4 expression and restored NFAT2 binding to IL-4 promoter –Knockdown of IL-4 abolished the analgesic effect of over-expression in NFAT2	–Paclitaxel increased AKAP150, decreased NFAT2, IL-10, IL-13, IL-4 levels, decreased calcineurin activity, and decreased interaction of NFAT2 with IL-4 –AKAP150 increased in response to paclitaxel and its knockdown reduced CIPN symptoms, increased calcineurin activity, and restored IL-4 levels –IL-4 decreased the enhanced action potentials within the DRG and reduced CIPN symptoms, and its downregulation contributed to enhanced CIPN symptoms –Increased NFAT2 reduced CIPN symptoms, potentially through restoring IL-4 levels –Regulation of IL-4 via the calcineurin/NFAT2 pathway mediated by AKAP150 (the decreased CN activity) inhibited the nuclei import of NFAT2 and the decreased NFAT2 reduced the IL-4 expression and participated in paclitaxel- induced neuropathic pain. Thus, up-regulated AKAP150 after paclitaxel injection was involved in neuropathic pain through inhibiting the enzyme activity of calcineurin, which might modulate the translocation of NFAT2 in the above conditions
Thibault et al. (2014)	Male Sprague Dawley rats* 123 total* –57 vincristine vs. 30 vehicle controls –6 Vincristine-oxycodone-saclofen treated –6 Vincristine-oxycodone-saline treated –6 Vincristine-saline-saclofen treated –6 Vincristine-saline-saline treated –6 Vincristine-morphine-saclofen treated –6 Vincristine-morphine-saline	–vincristine 0.1 mg/kg/day intraperitoneally for 2 five-day cycles with a two-day pause between cycles –Behavioral tests were performed on days 1 and 15 of vincristine treatment –The study of oxycodone and morphine effects was performed on days 15 and 19 (chronic)	–Vincristine-treated rats displayed increased static mechanical allodynia, hyperalgesia, and dynamic mechanical allodynia in comparison to baseline and saline-treated rats	–*Interventions* –Oxycodone intraperitoneally –Morphine intraperitoneally –Saclofen (GABA_B_ receptor antagonist) intrathecally –A single morphine or oxycodone injection reversed static mechanical allodynia, hyperalgesia and dynamic mechanical allodynia ◦ oxycodone was more effective than morphine to reduce static mechanical analgesia ◦ oxycodone reversed dynamic mechanical hyperesthesia but morphine only attenuated it –At the end of the analgesic chronic treatment on day 19, only oxycodone was able to maintain the analgesic effect on mechanical sensitivity –Following oxycodone treatment, 3 genes regulating receptor activity were observed in the small diameter DRG neurons, as well as their terminals in superficial laminae of the dorsal horn: Gabbr2 (GABA_B_2 receptor), Gabrb3 (GABA_A_ R subunit β3) and Gabrg1 (GABA_A_ R subunit γ1) –The analgesic effect of oxycodone on static mechanical allodynia was completely blocked by Saclofen, whereas its analgesic effect on mechanical hyperalgesia was only partially blocked	–Vincristine increased static mechanical allodynia, hyperalgesia, and dynamic mechanical allodynia –Oxycodone had longer lasting analgesic effects than morphine on vincristine-treated animals –Oxycodone only caused an upregulation of various GABA receptor transcripts in the DRG –The relieving effects of oxycodone, but not morphine, were either partially or completely blocked by GABA_B_ receptor antagonist
Yadav et al. (2015)	Male Sprague Dawley rats	–Paclitaxel 2 mg/kg intraperitoneally on 4 alternate days (days 1, 3, 5 and 7)	–Paclitaxel caused thermal hyperalgesia and mechanical allodynia –Paclitaxel decreased GABAergic inhibition in the dorsal horn in comparison to vehicle rats –Paclitaxel increased GAT-1 (presynaptic and astrocytic GABA transporter) and decreased GAT-3 (astrocytic GABA transporter) expression in the dorsal horn –Paclitaxel increased GABA uptake	–*Intervention*s –Intrathecal NO-711 (GAT-1 inhibitor) –Intrathecal SNAP5114 (GAT-3 inhibitor) –The paclitaxel-induced GABAergic suppression was alleviated by blocking GAT-1 but not GAT-3 –The thermal hyperalgesia and mechanical allodynia were significantly reversed by blocking GAT-1 but not GAT-3	–Paclitaxel induced thermal hyperalgesia and mechanical allodynia, decreased GABA signaling, and increased GABA uptake in the dorsal horn –Paclitaxel increased GAT-1 expression, and decreased GAT-3 expression in the dorsal horn –Blocking GAT-1 decreased the paclitaxel-induced GABA suppression and CIPN symptoms. These results were not observed with GAT-3 blockage

Overall, we identified four themes: brain hyperactivity, reduced GABAergic inhibition, inflammation, and GPCR/MAPK signaling. We discuss how these themes are supported by the studies and their implications for clinical research and ultimately treating and preventing CIPN in patients.

#### Hyperactivity

Neurotoxic chemotherapeutic agents lead to chronic hyperactivity within specific brain regions or as an overall state of the CNS. Hyperactivity was detected in the S2 and insula following oxaliplatin using fMRI to measure brain activity in response to cold stimulation of the tail (Nagasaka et al., 2017). Oxaliplatin produced hyperactivity in the somatosensory, cingulate, and motor cortices as measured by increased *p*-Erk-IR neurons, a marker of neuronal activity (Thibault et al., 2012). Finally, oxaliplatin produced hyperactivity as measured by field excitatory post synaptic potentials measured in post-mortem ACC slices (Nashawi et al., 2016).

These studies investigated different mechanisms underlying increased brain activity. For instance, voltage-gated potassium channels cause hyperpolarization, thereby requiring an increased stimulus for a neuron to fire an action potential. Oxaliplatin downregulates expression of potassium channel Kv2.2 in the somatosensory cortex, thereby requiring a smaller stimulus for neuronal firing (Thibault et al., 2012). Moreover, experimentally downregulating Kv2.2 in the somatosensory cortex (thereby removing the inhibitory mechanism) caused hyperactivity and CIPN symptoms in chemotherapy-naïve rats (Thibault et al., 2012). This is consistent with another study in rodents showing that systemic K^+^ channel knockout causes mechanical and cold sensitivity (Castellanos et al., 2020). Hyperactivity might also be caused by changes in voltage-gated sodium channel expression, as shown in the ACC in response to paclitaxel (Masocha, 2016), or by a reduction in GABA, an inhibitory neurotransmitter and the topic of the next theme (Nagasaka et al., 2017; Nashawi et al., 2016).

Neurotoxic chemotherapy also produced hyperexcitability in neurons of the spinal cord and the DRG, which bridge the peripheral nervous system and the spinal cord. One study found an increase in P2X7R-dependant glutamate release from cerebrocortical synaptosomes following oxaliplatin treatment; glutamate is the major excitatory CNS neurotransmitter, and its release was eliminated by P2X7R antagonists delivered to the spinal cord (Mannelli et al., 2015). A more positive resting membrane potential and a greater frequency of firing (i.e., hyperactivity) was observed in SOM^+^ excitatory interneurons in the outer lamina of the dorsal horn following paclitaxel treatment; experimentally blocking the IL-17 receptor reduced both hyperactivity and CIPN symptoms (Luo et al., 2019).

Taken together, CNS hyperactivity occurs in CIPN and correlates over time with changes in CIPN symptoms. Hyperactivity in the PAG, thalamus, ACC, and somatosensory cortex might be one of the final common pathways to symptoms of CIPN, as these brain regions support interoception (Kleckner et al., 2017), the processing and perception of sensations from the body (Craig, 2002; Khalsa et al., 2018). It seems plausible that brain amplification of peripheral inputs (i.e., hyperactivity) could help explain CIPN symptoms of hyperalgesia, an increased sensitivity to pain, and allodynia, the experience of pain to normally non-painful stimuli. Clinically, because brain hyperactivity is easy to measure during fMRI scanning or EEG recording, it could serve as an objective biomarker for CIPN used for diagnosis or as an endpoint in a clinical trial (i.e., a surrogate or target for treatment). Future research can explore whether specific patterns of brain hyperactivity (e.g., in the PAG vs. thalamus vs. insula vs. ACC) can help distinguish different subtypes of CIPN that might respond differently to different treatments or predict different natural histories of symptom escalation and recovery.

#### Decreased GABAergic Inhibition

A reduction in GABA levels in the brain may contribute to CIPN by decreasing inhibitory signaling, whereas experimental activation of the GABAergic system reduces and/or reverses CIPN symptoms. A decrease in GABA in the thalamus and dorsolateral PAG (dl-PAG) was reported after oxaliplatin treatment in rats that was accompanied by signs of CIPN (Ferrier et al., 2015; Xu et al., 2018). One study experimentally activated GABA_A_ receptors specifically in the dl-PAG, which decreased CIPN symptoms and reduced PAG hyperactivity (Xu et al., 2018). Another paper experimentally activated the GABA_A_ receptor via injection of muscimol into the insula/S2, which reversed hyperactivity in the insula/S2 during cold-stimulation as measured by fMRI and reduced CIPN symptoms in oxaliplatin-treated monkeys (Nagasaka et al., 2017). Similarly, one study showed that a bath application of GABA or a GABA_B_ receptor agonist in rats *ex vivo* ACC slices attenuated the increase in field excitatory post synaptic potentials, thereby reducing hyperactivity (Nashawi et al., 2016). GABAergic mechanisms in the brain have also been shown to mediate effects of compounds that reduce CIPN pain, as antagonizing the GABA_B_ receptor in the brain via ICV injection blocked the analgesic effects of CDP-Choline (Kanat et al., 2013).

Alterations in GABAergic signaling in the spinal cord may also play a role in CIPN. Transplant of GABA-producing cells within the spinal cord reversed paclitaxel-induced CIPN symptoms in mice with an intact GABA transporter but not in mutant mice missing a GABA-releasing transporter (Braz et al., 2015). GABA receptor activity was reduced and GABA transporter expression levels increased in the dorsal horn of paclitaxel-treated mice, suggesting increased GABA reuptake in the dorsal horn and decreased GABAergic inhibition (Yadav et al., 2015). Inhibiting the GAT-1 transporter, thereby allowing further release of GABA and enhancing neural inhibition, reduced paclitaxel-induced neuropathic pain. Therefore, directly increasing GABA levels or stimulating the GABA receptors enhanced inhibitory signals and eliminated CIPN symptoms. GABAergic pathways in the spinal cord are also involved in the treatment of CIPN symptoms by analgesics. Specifically, oxycodone, which upregulated GABA receptor mRNA in the DRG, had longer lasting analgesic effects than morphine in rats with vincristine-induced CIPN (Thibault et al., 2014). Oxycodone-induced analgesic effects were eliminated by a GABA_B_ receptor antagonist, further suggesting that GABA is required for oxycodone to reduce CIPN symptoms. Lastly, injecting pro-inflammatory IL-17 into the spinal cord also decreased inhibitory signals that GABA produced, indicating that inflammation might be an upstream pathway for the inhibition of GABA (Luo et al., 2019).

Taken together, attenuation of inhibitory mechanisms could explain the observed increase in excitatory signals and increased brain activity observed in CIPN. Restoration of GABA levels specifically in the brain or non-specifically in the spinal cord and brain can reduce CIPN symptoms, and multiple analgesic drugs for CIPN control balance of inhibitory and excitatory transmission. Clinically, GABA and GABA analogs have been investigated as potential analgesics in CIPN and other conditions (Zaręba et al., 2020) because GABAergic neurons and receptors are involved in coordination of the perception and response to noxious stimuli (Enna and McCarson, 2006). However, GABAergic drugs are not commonly used as analgesics given their side effects such as muscle weakness, drowsiness, fatigue, upset stomach, and nausea (Enna and McCarson, 2006). Therefore, although it may not be desirable to introduce exogenous sources of GABA in the human brain to treat CIPN, non-invasive measurement of GABA in the brain (Mullins et al., 2014) might help direct the development and optimization of non-invasive interventions to maximize GABAergic signaling through endogenous mechanisms. Also, GABA levels in the brain could serve as a biomarker for CIPN or a mechanistic endpoint for clinical trials to treat or prevent CIPN.

#### Neuroinflammation

Neuroinflammation has been frequently proposed as an underlying peripheral mechanism of CIPN development (Wang et al., 2012; Starobova and Vetter, 2017; Ma et al., 2018; Brandolini et al., 201f9). Studies from our review suggest that the CNS is also in a pro-inflammatory state during CIPN, and that mitigating the increased neuroinflammation alleviates symptoms of CIPN. Indeed, oxaliplatin increases the levels of pro-inflammatory cytokines and their receptors within the dl-PAG, and decreases GABA levels within the dl-PAG (Xu et al., 2018). When Xu et al. injected pro-inflammatory cytokine receptor antagonists specifically to the dl-PAG to block inflammation, CIPN symptoms were reduced and GABA levels were restored.

Increases in both CIPN symptoms and pro-inflammatory markers in the spinal cord are observed following chemotherapeutic treatment. For instance, paclitaxel treatment increased the level of the pro-inflammatory cytokine IL-17 in the spinal cord (Luo et al., 2019). Intrathecal injection of IL-17 resulted in CIPN symptom development and decreased GABA signaling. IL-17 also increased neural excitation by increasing the amplitude of NMDAR excitatory post synaptic currents and the firing frequency of excitatory interneurons. Knockdown of the IL-17 receptor in the spinal cord eliminated all these effects, reducing CIPN, restoring GABA, and reducing hyperactivity (Luo et al., 2019). Increases in TNF-α, another pro-inflammatory cytokine, were also observed in the spinal cord following oxaliplatin treatment concomitant with CIPN symptoms. Moreover, experimentally blocking nuclear receptors REV-ERBs, upstream regulators of inflammatory gene transcription, in the spinal cord prevented LPS- and TNF-α-induced transcription of pro-inflammatory cytokines IL-1β and IL-6, and reduced CIPN symptoms (Morioka et al., 2019). Finally, neurotoxic agents such as paclitaxel increased pro-inflammatory cytokines and reduced levels of anti-inflammatory cytokines IL-4, IL-10, and IL-13. Restoration of IL-4 in the spinal cord decreased neuronal hyperactivity and attenuated CIPN symptoms (Nie et al., 2018).

Thus, several lines of evidence implicate neuroinflammation in the brain and spinal cord in both the development and maintenance of CIPN and suggest that anti-inflammatory treatments at the level of the CNS suppress CIPN. Chronic CIPN symptoms/maintenance may be mediated by the brain’s neuroinflammatory state based on similar findings in chronic pain (Ji et al., 2014; Ji et al., 2018). Indeed, new evidence suggests that glial activation may lead to long term alterations in neuronal excitability and maintain pain sensation even after the original insult has receded (Hansson, 2010; Ji et al., 2018). Reducing neuroinflammation may consequently help alleviate both acute and chronic CIPN. These observations raise the possibility that CIPN might be treated by interventions that reduce neuroinflammation, such as drugs (e.g., non-steroidal anti-inflammatory drugs shown helpful for diabetic neuropathy Cohen and Harris, 1987) or behavioral interventions (e.g., exercise Gleeson et al., 2011; Kleckner et al., 2018; Kleckner et al., 2019).

#### GPCR/MAPK

Signaling cascades downstream of binding to GPCRs are also implicated in CIPN (Borroto-Escuela et al., 2017). Paclitaxel CIPN symptoms were reduced using genetic knock out mice or antagonizing one or both kinin B_1_ and B_2_ GPCRs either systemically (intraperitoneally) or only centrally (ICV), suggesting a role of these GPCRs in the CNS in modulating CIPN (Costa et al., 2011). ICV administration of Orexin-A, a neuropeptide working through a GPCR, produced antinociceptive effects in mouse models of CIPN symptoms, whereas antagonizing the receptor blocked the orexin-induced antinociception (Toyama et al., 2017). Antinociceptive effects of morphine and oxycodone in oxaliplatin-treated rats (Kanabara et al., 2014a; Kanabara et al.,2014b) were blocked by ICV administration of either a G_i/o_ protein receptor inhibitor, or a GIRK1 channel blocker, which is downstream of the G_i/o_ protein. Elevated levels of phosphorylated PKC, MAPK, ERK, and SAP/JNK were also observed specifically within the thalamus and the PAG tissue homogenates following oxaliplatin treatment (Sanna et al., 2016; Norcini et al., 2009). These changes, along with CIPN symptoms, were reversed upon administration of a PKC inhibitor to the brain via ICV injection.

Similarly, other studies reported increased ERK phosphorylation in the spinal cord following oxaliplatin injection (Zhang et al., 2019). Furthermore, injection of an ERK inhibitor both reduced the level of phosphorylated ERK and reversed CIPN symptoms. No change in phosphorylation levels of other MAPKs, p38 or JNK was observed, which contrasts with the observed role of MAPK in the brain in CIPN (Sanna et al., 2016; Norcini et al., 2009). More work is necessary to elucidate the impact of chemotherapy on the kinetics of such signaling cascades and how these mechanisms can be exploited for treatment of CIPN.

There are several implications of these findings. First, GPCR/MAPK signaling in the brain appears to be involved in CIPN. Second, multiple drugs that reduce CIPN modulate MAPK signaling pathways. Because these signaling pathways are ultimately responsible for the transcription and synthesis of various proteins, it is difficult to discern the exact changes occurring (as is also the case with inflammation). However, GPCRs have become the focus of research attention for treating multiple other brain-mediated conditions such as anxiety and depression (Borroto-Escuela et al., 2017), and therefore may be promising targets of treatment for CIPN as well.

#### Other Findings

Several brain intervention studies did not fit into one of the above themes. Two lines of evidence support a role for the cholinergic system in the CNS in contributing to CIPN. First, oxaliplatin both increased the expression of the M2 acetylcholine receptors and decreased acetylcholine levels in the posterior insula whereas either activating the M2 receptor or restoring acetylcholine levels specifically in the posterior insula reversed CIPN symptoms (Ferrier et al., 2015). Second, inhibiting the nonselective nicotinic acetylcholine receptor and the α7 selective nicotinic acetylcholine receptor in the brain via ICV injection blocked the antinociceptive effects of CDP-Choline (Kanat et al., 2013). CDP-choline is an intermediate in the pathway for cell membrane phospholipid synthesis, and separates into its two components in the body, cytidine and choline. Choline is the primary precursor used by the brain to synthesize acetylcholine, suggesting that cholinergic pathways are involved in CIPN and its treatment. CDP-choline has also been shown to increase dopamine and norepinephrine levels in the CNS (Secades and Frontera, 1995; Secades and Lorenzo, 2006). These two monoamines, along with serotonin, have been shown to be involved in CIPN, as serotonin, norepinephrine, and dopamine reuptake inhibitors increased extracellular levels of their respective monoamines and each reversed a different combination of CIPN symptoms (Hache et al., 2015). Administration of a single triple monoamine reuptake inhibitor elevated the extracellular levels of all three monoamines, and completely reversed all CIPN symptoms. Similarly, systemic duloxetine (also a serotonin and norepinephrine reuptake inhibitor) reduced CIPN symptoms (Smith et al., 2013) consistent with Nagasaka et al. (2017). Third, ICV delivery of gabapentin, a voltage-gated Ca^2+^ channel inhibitor, decreased paclitaxel-induced mechanical hypersensitivity and pain aversiveness in a dose-dependent manner (Juarez-Salinas et al., 2018). Finally, injection of neuronal nitric oxide synthase into the lateral cerebral ventricle of paclitaxel-treated rats reversed CIPN symptoms through hyperbaric oxygen, and the antinociceptive effect of hyperbaric oxygen was eliminated with an inhibitor of neuronal nitric oxide synthase (Zhang et al., 2019). Taken together, these reports support diverse mechanisms of brain hyperactivity in CIPN and mechanisms to reduce that hyperactivity. These less commonly studied mechanisms suggest the presence of promising opportunities for future research.

## Overall Discussion

**FIGURE 2:**
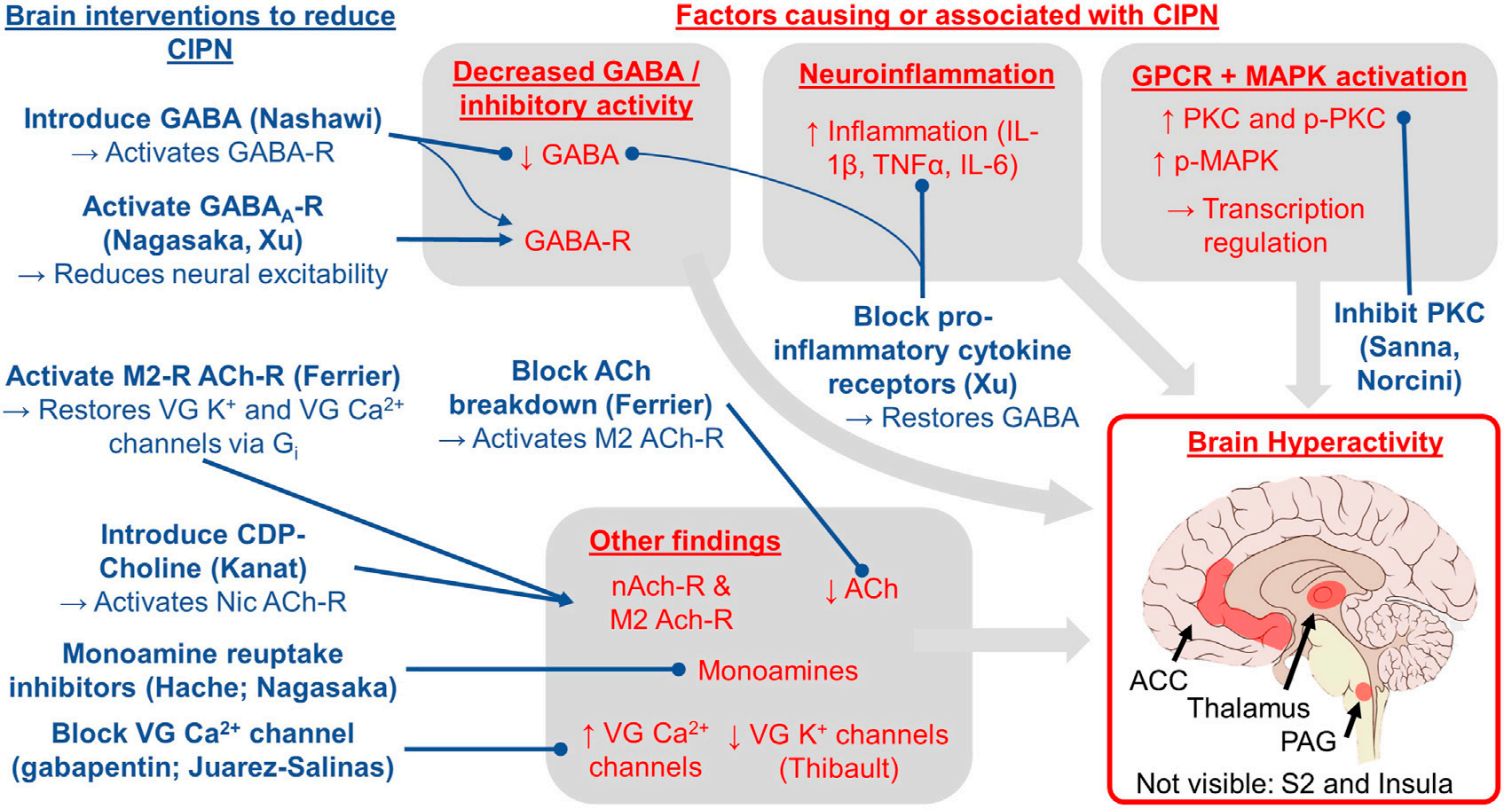
Conceptual model for the role of the brain in CIPN based on the evidence reviewed herein. The red text indicates brain factors that cause or are correlated with CIPN. The blue text indicates brain interventions shown to treat or reduce CIPN via the experimental studies (first author provided in parentheses; all studies reviewed in Table 3). Lines ending in a circle indicate blocking or reducing the target whereas lines ending in an arrow indicate activating or increasing the target. The key brain regions studied and implicated in our review include the periaqueductal gray (PAG), thalamus, anterior cingulate cortex (ACC), secondary somatosensory cortex (S2), and insula.

*Summary of findings* (Figure 2). This is the first paper to summarize the literature on the role of the brain in CIPN. We reviewed five correlational studies of CIPN and brain imaging in humans and non-human primates (Table 2), 16 papers studying interventions to the brain that cause or reduce CIPN symptoms (Table 3), and eight papers using interventions to the spinal cord (Table 4) and we highlight four key themes. First, CIPN is associated with hyperactivity and hyperexcitability in several brain regions including the PAG, thalamus, ACC, S2, and insula, which makes sense as they are part of well-known circuitry related to sensation and perception including pain (Kleckner et al., 2017; Reddan and Wager, 2018). Second, CIPN is associated reduced GABAergic inhibition in the brain, thereby changing excitatory/inhibitory balance to create a molecular environment promoting neuronal hyperactivity. Moreover, activating GABA receptors or increasing GABA levels reduces symptoms of CIPN. Third, these brain regions exhibit a pro-inflammatory state, which is consistent with prior work indicating that oxaliplatin activates astrocytes in the ACC in mice (Masocha, 2015) and that neuroinflammation increases neural excitability (Leung and Cahill, 2010). Blocking key inflammatory pathways in the brain restores GABA levels, reduces neuronal excitability, and reduces CIPN. Fourth, GPCR and MAPK phosphorylation pathways are also implicated in CIPN, which lead to changes in transcription and neuroinflammation (and likely other changes). Experimentally manipulating the GPCR pathways to reduce PKC or MAPK phosphorylation in the brain reduces CIPN symptoms. Finally, studies suggest CIPN is related to monoamines (e.g., serotonin, norepinephrine, dopamine), oxidative stress, acetylcholine receptor expression, and ion channel expression (reduction in voltage-gated K^+^ and increase in voltage-gated Ca^2+^ channel activity).

Our review has the potential to shift the theoretical paradigm of CIPN as not exclusively a peripheral phenomenon and help focus more research attention on the brain. This shift can help advance preclinical and clinical research on CIPN to inform additional and more impactful studies of the brain in CIPN, which are urgently needed according to the 2017 National Cancer Institute Clinical Trials Planning Meeting on CIPN (Dorsey, Kleckner et al., 2019). This future research agenda will ultimately lead to a greater understanding of CIPN and more effective diagnostics, prophylactics, and treatments for CIPN. In the remaining paragraphs, we discuss potential mechanisms of how chemotherapy affects the brain, how the brain is involved in CIPN at the neural systems level, implications for the role of the brain in CIPN for preclinical research, clinical research, and clinical treatment of CIPN, the potential for a unified theory of the brain in multiple chemotherapy toxicities, and finally, we address the strengths and limitations of our review. We consider changes in the brain at various levels of analysis (e.g., molecular, cellular, systems/networks) for two reasons: (1) because most brain measures occur at the microscopic level in non-human animals yet at the macroscopic level in humans, and (2) to consider both reductionist and holistic approaches to relationships between neurobiology and subjective experience (i.e., symptoms) (Krakauer et al., 2017).

*The possibility of direct and indirect effects of chemotherapy on the brain in CIPN*. It is possible but unlikely that brain changes summarized here (e.g., hyperactivity) are caused by chemotherapy entering the brain. Indeed, the idea that chemotherapy accumulates in the human brain has been debated, and likely depends on the type of chemotherapy, the dose density, and other factors that may compromise the blood brain barrier (Branca et al., 2018). In rodents, although paclitaxel has been found in the brain after peripheral infusions (Cavaletti et al., 2000), cisplatin has only been found in the brain under extreme circumstances such as excessive chemotherapy dose (Screnci, McKeage et al., 2000), hypoxia, or lipopolysaccharide challenge (Minami et al., 1996a; Minami et al., 1996b; Minami et al., 1996c; Minami et al., 1998). There is relatively more evidence that the spinal cord accumulates neurotoxic chemotherapy, such as oxaliplatin found in the cerebrospinal fluid (Huang et al., 2016) and DRG in humans (Krarup-Hansen et al., 1999) and rodents (Screnci et al., 2000), perhaps because the DRG lack the protective blood brain barrier.

It appears more likely that brain changes seen in CIPN are caused by indirect effects of neurotoxic chemotherapy on the brain. Indeed, we hypothesize that the brain undergoes significant compensation due to altered afferent input including unusually excessive input from some sensory nerves and lack of input from others, as is the case with phantom limb pain (Makin and Flor, 2020). Brain compensation makes sense from a predictive coding perspective of the brain (Friston, 2018) (and related ideas such as active inference and the Bayesian brain hypothesis), which posits that perceptual experience is driven primarily by the brain’s *predictions* of a given moment of consciousness, and that prediction is merely tailored—not driven—by afferent sensory input. Note that predictive coding models of the brain contrast traditional stimulus-response models of the brain, in which perceptual experience is primarily driven by sensory input. In accord with a modern neuropsychological perspective of the mind (Barrett, 2017; Hutchinson and Barrett, 2019), we consider perception to include sights, sounds, feelings, emotions, thoughts, memories, and symptoms, including those of CIPN, as we have previously suggested (Kleckner et al., 2018). Therefore, neurotoxic chemotherapy might cause the brain’s circuitry for generating predictions (and thus perceptions) to undergo significant changes in neural coding to account for the chronically unexpected peripheral sensory input that occurs in CIPN. Changes in coding would likely require additional metabolic needs to brain regions involved in predictions, consistent with brain hyperactivity and hyperperfusion observed in this review. Indeed, the regions of the brain that are proposed to initiate predictions include the major hubs of the DMN such as the ACC (Barrett and Simmons, 2015), which are highly connected to sensory regions such as the insula, S2, thalamus, and PAG (Kleckner et al., 2017), which are all implicated in CIPN per our review herein. This hypothetical compensation in the brain might have implications for traditional perspectives of the role of the brain in pain, such as how neurotoxic chemotherapy might cause a reduction in descending inhibition of pain (e.g., according to Gate Control Theory Melzack and Wall, 1965). For completeness, these hypothetical changes in the brain would be concurrent with other effects of chemotherapy such as neuroinflammation (McLeary et al., 2019), which sensitizes neurons and causes hyperactivity (Vezzani and Viviani, 2015).

*Preclinical research implications* First and foremost, we need more studies testing brain interventions and brain mechanisms because this type of detailed work is unethical or impractical in humans. Indeed, chemotherapy is never delivered to humans in the absence of cancer and an impact of prior cancer cannot be excluded; thus, animal models uniquely allow us to learn about CIPN in the absence of cancer. Also, we should not assume that what we observe in the periphery or spinal cord in relation to CIPN also occurs in the brain, because the brain, the spinal cord, and peripheral nervous system differ in terms of their function, their biology, their accessibility by neurotoxic drugs, etc. Second, we need more studies of animals whose brains and CIPN more closely resemble that of humans (Hama et al., 2018), and the animal research should use measures and analytical methods more similar to those used in human studies. In terms of measures, to our knowledge there are only two studies in rodents using structural, functional, or diffusion tensor MRI (Ferris et al., 2019; Alkislar et al., 2020), which are measures commonly used in humans. In terms of analytical methods, many human brain imaging studies assess correlations between brain measures or their changes (e.g., activity, perfusion) and CIPN measures or their changes, but this type of analysis is only rarely conducted in the preclinical studies we identified. Preclinical studies typically randomize animals to chemotherapy vs. vehicle (which is good), demonstrate that CIPN is present in the chemotherapy group (which is also good), and assess brain differences between CIPN and non-CIPN groups (which is insufficient). The latter analysis is insufficient because brain differences may not be related to CIPN but rather reflect brain differences attributable to other chemotherapy effects (e.g., chemotherapy-induced cognitive impairment, changes in food consumption, hydration status, voluntary physical activity, social behavior, etc.). Next, researchers should recognize that possible CIPN interventions can affect the brain (i.e., CNS penetrant vs. peripherally restricted), even if the drugs are delivered systemically (e.g., Slivicki et al., 2018; Slivicki et al., 2019). Finally, considering the role of the brain could provide insight into the autonomic components of CIPN (Verstappen et al., 2003; Nahman-Averbuch et al., 2014). For example, peripheral neuropathy often involves dysfunction in the reflexive wrinkling of glabrous skin during water immersion (Ng et al., 2013; Wilder-Smith, 2015), which appears to be related to central autonomic function (Win et al., 2010), which is mediated by the brain (Sklerov et al., 2019).

*Clinical research implications: brain mechanisms, brain biomarkers, and brain-based interventions* First, regarding brain mechanisms, out of the four common themes we identified, only one of those has been investigated in humans (hyperactivity), and the remaining themes should be studied to gain a better understanding of the role of the human brain in CIPN. Second, knowledge of those brain mechanisms can inform brain biomarkers of CIPN, for which there are currently none. If a highly accurate and reliable biomarker for CIPN is identified, it can help increase sensitivity and reduce bias in clinical trials of CIPN treatments, and it might also serve as a risk factor for predicting which patients will experience the worst CIPN or experience the best recovery of CIPN symptoms after completion of chemotherapy. In addition, the biomarker might be related to the brain but could be measured peripherally, such as a recent study finding that serum levels of brain-derived neurotrophic growth factor predicted CIPN and overall survival in 91 patients with multiple myeloma receiving bortezomib and/or thalidomide (Szudy-Szczyrek et al., 2020). Third, an impressive array of brain-based interventions warrant testing for their ability to modulate brain mechanisms involved in CIPN. Those interventions include neuromodulation (rTMS, tDCS, spinal cord stimulation), neurofeedback (fMRI, EEG per Prinsloo et al. in Table 2), and pharmacological, behavioral, and peripheral interventions that also affect the brain such as neurotransmitter modulators (e.g., duloxetine, bupropion), peripheral nerve stimulation (transcutaneous electrical nerve stimulation, scrambler therapy, vagus nerve stimulation), meditation, exercise, and cognitive behavioral therapy or combination therapies to amplify an intervention’s effect on the brain (e.g., tDCS during exercise, duloxetine plus exercise). In future studies of those interventions, it is important to include brain measures to help elucidate brain mechanisms of treatment or potential subgroup effects (responders vs. non-responders).

*Clinical implications* Additional knowledge of the role of the brain in CIPN can ultimately inform better CIPN diagnostics, biomarkers, and treatments. Brain imaging might help inform a diagnosis of CIPN or identify CIPN sub-types, which has been successful in other brain-mediated conditions such as depression (Sanacora et al., 2004; Takamura and Hanakawa, 2017; Tokuda et al., 2018). These biomarkers could help track toxicity during chemotherapy to help patients and medical oncologists weigh the risk/benefit ratio and decisions of chemotherapy dose vs. risks of long-term CIPN toxicity and cancer treatment effectiveness. The biomarkers could also help track response to interventions to reduce CIPN to determine that they are working via the expected mechanism and to select the proper dose of an intervention (e.g., amount of drug, intensity of neurostimulation or neurofeedback, amount or type of exercise).

*A unified brain-based theory could explain multiple chemotherapy-induced toxicities* including CIPN, fatigue, distress, nausea, and cognitive impairment. This idea leverages modern neuroscientific theories that emotions and other mental states are derived from interactions among a finite set of brain processes (Barrett, 2017; Kleckner et al., 2017; Barrett and Satpute, 2019; Hutchinson and Barrett, 2019). One of the most important brain processes is interoception (Craig, 2002; Khalsa et al., 2018), the processing of bodily sensations. In our prior work, we delineated an interoceptive brain system, which includes the insula, ACC, somatosensory cortex, thalamus, and PAG (all implicated in CIPN), as well as other regions largely delineated by a DMN-like network and a sensory-oriented network (Kleckner et al., 2017). Interoception likely plays a role in multiple chemotherapy toxicities because so many chemotherapy toxicities are strongly embodied with somatic symptoms. For example, fatigue is related to rationing energy resources (i.e., allostasis, which is intimately linked to interoception (Kleckner, Zhang et al., 2017)); fatigue has also been associated with CIPN in multiple studies (e.g., (Mols et al., 2013; Eckhoff et al., 2015; Beijers et al., 2016; Bonhof et al., 2019)). Distress, including anxiety and depression, is often experienced somatically in the heart, lungs, and gut, and distress and emotional processing are strongly dependent upon interoception (Paulus and Stein, 2010; Kleckner, Zhang et al., 2017). In addition, distress has been associated with CIPN (Bao et al., 2016; Toma et al., 2017; Lee et al., 2018; Bonhof et al., 2019); in fact, a recent study in 471 survivors of colorectal cancer found that symptoms of distress (anxiety, depression) mediate the effects of CIPN on fatigue (Bonhof, van de Poll-Franse et al., 2019). Nausea is based on predictions on the state of the gut and relies heavily on interoception (Wickham, 2020), and has been associated with CIPN (Mols et al., 2013; Ezendam et al., 2014). Finally, cognitive impairment includes memory components, the encoding of which includes bodily sensations, and thus interoception (Terasawa and Umeda, 2017). Cognitive impairment has been associated with CIPN in rodents (Fardell et al., 2015), but research in humans has been limited. There may be multiple underlying mechanisms contributing to a unified account of the role of the brain in chemotherapy toxicities (e.g., neuroinflammation Vichaya et al., 2015). However, because these ideas are relatively new and understudied, additional studies need to be designed to test the possibility and utility of a unified brain-based theory of multiple chemotherapy toxicities.

If our hypothesis is false, and the brain does not play a prominent or causal role in the development and treatment of CIPN, then there are alternative implications for future work. First, CIPN interventions that affect the brain (e.g., duloxetine) could merely mask symptoms of CIPN rather than treating factors that are part of the pathophysiological mechanism per se. Second, it suggests that researchers should continue to focus on mechanisms and treatments for peripheral damage to stop CIPN at its peripheral source. However, the lack of successful treatments for CIPN thus far despite significant research suggests that researchers should include more work with animal models that better translate to CIPN in humans (e.g., macaques Hama et al., 2018).

*Strengths of this review* This is the first review to summarize evidence regarding the contribution of the brain to CIPN and to summarize implications for research and treatment of CIPN. This review is also very timely given that a recent meeting of CIPN experts at the National Cancer Institute stressed the urgency and importance of developing new theoretical frameworks to understand CIPN (Dorsey et al., 2019). Second, our work is innovative in that our synthesis of results leverages modern neuroscience perspectives on mental states (e.g., the role of interoception and predictive coding). This novel theoretical framework of CIPN supports an innovative set of hypotheses regarding the role of the brain in CIPN and perhaps other chemotherapy toxicities. This framework will advance future research and ultimately clinical treatments for patients receiving chemotherapy. Third, multiple papers from different independent research groups support our hypothesis that the brain plays a prominent role in CIPN. In fact, these results are remarkably consistent with one another in terms identified themes, and there were multiple partially overlapping consistencies (e.g., activating the GABA receptor reduced CIPN whether by introducing a GABA-R agonist, increasing GABA levels, or blocking inflammation pathways to increase GABA).

*Limitations of this review* Due to heterogeneity in methods such as chemotherapy type, chemotherapy dosing schedule, brain measures, brain interventions, and CIPN assessments (e.g., cold allodynia, mechanical allodynia, mechanical hypersensitivity, and the various tests thereof), the emerging literature in this area reflects only a small number of papers supporting each theme. Second, our review does not include all possible explanations on the role of the brain in CIPN and there are likely other factors involved that simply have not been studied yet. Moreover, some of the brain-based interventions might also affect other regions of the brain or body, and perhaps systemically, or vice versa (e.g., if the delivered drug goes from the brain to the periphery, or indirect effects of a reduction in neuroinflammation reducing peripheral inflammation). Third, all the evidence suggesting that the brain plays a prominent or causal role in CIPN is based in non-human animals, and these methods are unethical in humans. However, even given these limitations, our review is important because it highlights gaps in the literature and opportunities for future research to further test the contributing role of brain mechanisms to CIPN.

*Conclusion* The vast majority of research on CIPN has focused on peripheral nerve damage but has yet to produce significant advances in the prevention and treatment of CIPN despite nearly 100 clinical trials for CIPN (Hershman et al., 2014; Loprinzi et al., 2020). Herein, we investigated the hypothesis that the brain plays a prominent or even causal role in CIPN by reviewing the literature on experimental manipulations of the brain to see its effect on CIPN in non-human animals. Our review implicated four common themes related to the role of the brain in CIPN, with brain hyperactivity being a key feature of the pathology of CIPN. We identified specific implications for preclinical research, clinical research, and clinical diagnosis, prevention, and treatment of CIPN that leverages knowledge of the role of the brain in CIPN. We also set the stage for a powerful unified brain-based theoretical framework for multiple chemotherapy toxicities, which is the first theory of its kind to our knowledge. Our review is the first to investigate the role of the brain in CIPN and it paves the way for more brain-based research, more advanced and specific theories on the role of the brain in CIPN, and clinical applications to prevent and treat CIPN to ultimately reduce the burden of chemotherapy on patients with cancer.

## Footnotes

1 To easily find papers in the alphabetically sorted tables, we refer to each paper by the last name of the first author.

2 The Prinsloo paper used a waitlist control which cannot account for non-specific effects of neurofeedback, as opposed to a sham neurofeedback procedure.
